# Quality Improvement and Patient Safety Rounds—A New Paradigm in Cardiothoracic Surgery

**DOI:** 10.1016/j.atssr.2023.11.026

**Published:** 2023-12-12

**Authors:** Joel Bierer, Edgar Chedrawy, Christine Herman, Garrett Walsh, Monisha Sudarshan, Steven Harrington, Christopher Feindel, Susan Moffatt-Bruce, The Society of Thoracic Surgeons Workforce on Patient Safety

**Affiliations:** 1 Division of Cardiac Surgery, Department of Surgery, Dalhousie University, Halifax, Nova Scotia, Canada; 2 Department of Thoracic and Cardiovascular Surgery, University of Texas MD Anderson Cancer Center, Houston, Texas; 3 Division of Thoracic Surgery, Cleveland Clinic Foundation, Cleveland, Ohio; 4 Michigan Society of Thoracic and Cardiovascular Surgeons Quality Collaborative, Ann Arbor, Michigan; 5 Division of Cardiovascular Surgery, Peter Munk Cardiac Centre, University Health Network and the University of Toronto, Toronto, Ontario, Canada; 6 Lahey Hospital & Medical Center, Burlington, Massachusetts

## Abstract

**Background:**

The delivery of cardiothoracic health care is complex, and despite best efforts, adverse patient outcomes do occur. There is significant heterogeneity in morbidity and mortality rounds within and between institutions as well as undertones of a “blame and shame” culture prohibitive to meaningful quality assessment and improvement of patient care. The Quality Improvement and Patient Safety (QIPS) program was designed to address these issues.

**Methods:**

QIPS is built on the Donabedian model of quality and established phase-of-care adverse event analysis. It focuses on cultivating a culture of transparency. Every case review yields important demographic data, event factors, and the case inflection point, creating an institution-specific QIPS database. The QIPS format was presented by The Society of Thoracic Surgeons Workforce on Patient Safety at 2 Society of Thoracic Surgeons webinar series in December 2021 and August 2022. Descriptive data on both presentations were collected.

**Results:**

The December 2021 webinar had 75 unique viewers from 13 countries, and there were 343 asynchronous playbacks. The August 2022 webinar had 38 unique viewers from 9 countries and 310 asynchronous playbacks.

**Conclusions:**

The standardized QIPS methods allow consistent recording of event factors and the inflection point, which are root causes of case morbidity or mortality. Dedication to the program will build a granular databank and identify recurring individual or system issues to launch quality improvement initiatives that address institution-specific needs. QIPS is simple, effective, and reproducible and seeks to create a culture of patient-centered quality and excellence in cardiothoracic surgery programs.

*To Err Is Human: Building a Safer Healthcare System* was a landmark report that identified the critical need to evaluate medical errors and to enhance the quality of health care in America.^1^ It identified a substantial weakness in traditional morbidity and mortality (M&M) conferences: they are not able to address systemic issues based on any single case review.^1^ “Their value in improving the quality of care could be substantially increased if ongoing data are kept to identify repeated complications and time trends.”^1^ Despite this compelling report, a highly heterogeneous and inconsistent structure of M&M rounds or quality assurance programs remains within and between institutions, yielding inconsistent improvements in patient safety and quality of care.^2^^,^^3^

Cardiothoracic surgeons have an illustrious history of innovations in medical treatment, technology, leadership, and, importantly, quality of health care. Two years before the publication of *To Err Is Human*, the pioneering Northern New England Cardiovascular Disease Study Group disseminated their results on a statewide analysis of mortality after coronary artery bypass grafting with suggestions on improving postoperative outcomes in this population of surgical patients.^4^ More recently, the Michigan Society of Thoracic and Cardiovascular Surgeons developed the “phase-of-care” analysis to facilitate a reproducible structure of evaluating adverse events to precisely direct quality improvement initiatives.^5^ Therefore, a systematic approach to adverse event analysis has been well established in the cardiothoracic specialty.

However, inconsistent participation by surgeons in traditional M&M rounds remains, perhaps because of a perceived culture of blame, fear of threats to referral patterns, or compensation and practice restrictions, all of which significantly reduce the potential power of adverse event analyses.^6^^,^^7^ Quality of care reviews are most effective when there is universal participation from all surgical attendings.^5^^,^^8^ Consequently, the Accreditation Council for Graduate Medical Education and the Royal College of Physicians and Surgeons of Canada have committed to creating a training culture that emphasizes excellence in patient safety and quality of care in a culturally safe environment.^9^^,^^10^ The Quality Improvement and Patient Safety (QIPS) program embraces this call to action to facilitate multidisciplinary M&M reviews that are resident led and attending supported while cultivating a culture focused on transparent and constructive discussion to enhance the safety and quality of care for our patients. This instructional report aims to provide detailed methods on the QIPS framework in cardiothoracic surgery so that this tool can be adopted to facilitate culturally safe patient safety event analysis.

## Methods

### QIPS Program Objectives

**Figure 1:**
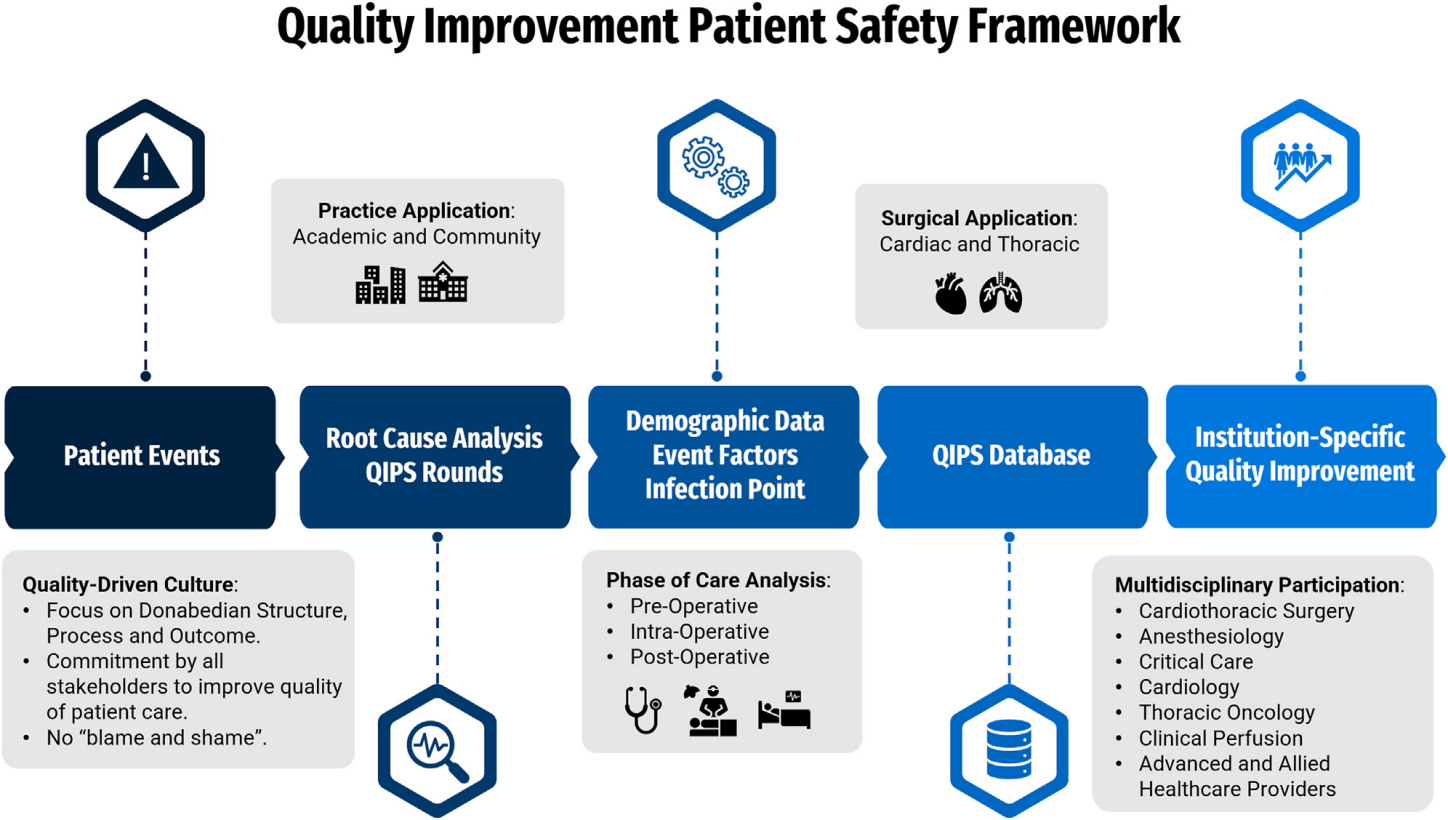
Quality Improvement and Patient Safety (QIPS) Framework.

The QIPS format aims to critically evaluate adverse events and to identify areas of improvement within cardiothoracic programs and the framework outlined in Figure 1. QIPS was initially designed in an academic setting, focusing on trainee participation, but the format is equally applicable to nonacademic centers.

The program has 4 primary objectives:

1. Develop cardiothoracic care provider’s critical thinking and professional skills.
2. Continuously evaluate adverse patient events in a consistent phase-of-care approach.
3. Launch data-driven and institution-specific quality improvement initiatives to enhance the quality of care for the patients we care for.
4. Cultivate a culture of awareness, transparency, collaboration, and quality in cardiothoracic divisions.

### QIPS Program Administration and Structure

The QIPS program should consist of 3 identifiable roles. First, the program or division chair, likely a cardiothoracic surgeon attending with quality improvement experience, is responsible for directing the program. Second, a program administrator schedules the QIPS rounds, maintains the central list of cases to be reviewed, assigns trainees for case presentations, and collects the completed QIPS data forms. Third, a data manager or analyst transfers the data derived from each case from the data form to the central QIPS database and conducts the statistical analyses. Outside these 3 roles, the cardiothoracic surgical group, resident program, fellowship program, and allied health professionals should participate as key stakeholders in this initiative.

### Case Selection for Review

The QIPS format enables the analysis of patients who have experienced a severe adverse event or fatality. For cardiothoracic surgery programs establishing the QIPS process at their institution, beginning with mortality reviews allows efficient implementation before including more challenging near-misses or morbidity events. Mortalities are unambiguous and routinely recorded at the divisional level and on The Society of Thoracic Surgeons (STS) case data form of participating institutions. It is imperative to have a reliable and standardized method of capturing all patient events to ensure a nonbiased data collection process. The program administrator records patient cases to be reviewed in an encrypted central database.

### QIPS Rounds Structure

Monthly sessions should be dedicated to QIPS rounds; the dates are prespecified at the beginning of each calendar year and rarely rescheduled. The timing should optimize the attendance of relevant stakeholders, such as cardiothoracic surgeons, trainees, anesthesiologists, intensivists, clinical perfusionists, cardiologists, oncologists, and nursing staff. Participation by the multidisciplinary team is crucial in having a thoughtful discussion and identification of patient care issues. Clinical and hospital leaders must espouse clear communication to all stakeholders of the timing and purpose of these rounds in every instance.

An introductory session on the QIPS methods and structure for institutions implementing the program is helpful for orientation, and this has been shown to enhance the quality of the case presentations and understanding of the structured format.^11^ Each QIPS session features the review of 4 patients, depending on the case complexity and the team’s experience with the QIPS format. One moderator, usually the program chair, quality director, or other attending cardiothoracic surgeon, should lead the rounds and record the multidisciplinary case discussions. A rotating schedule of moderators to include all cardiothoracic surgery attendings encourages participation.

### QIPS Case Presentation and Discussion

**Figure 2:**
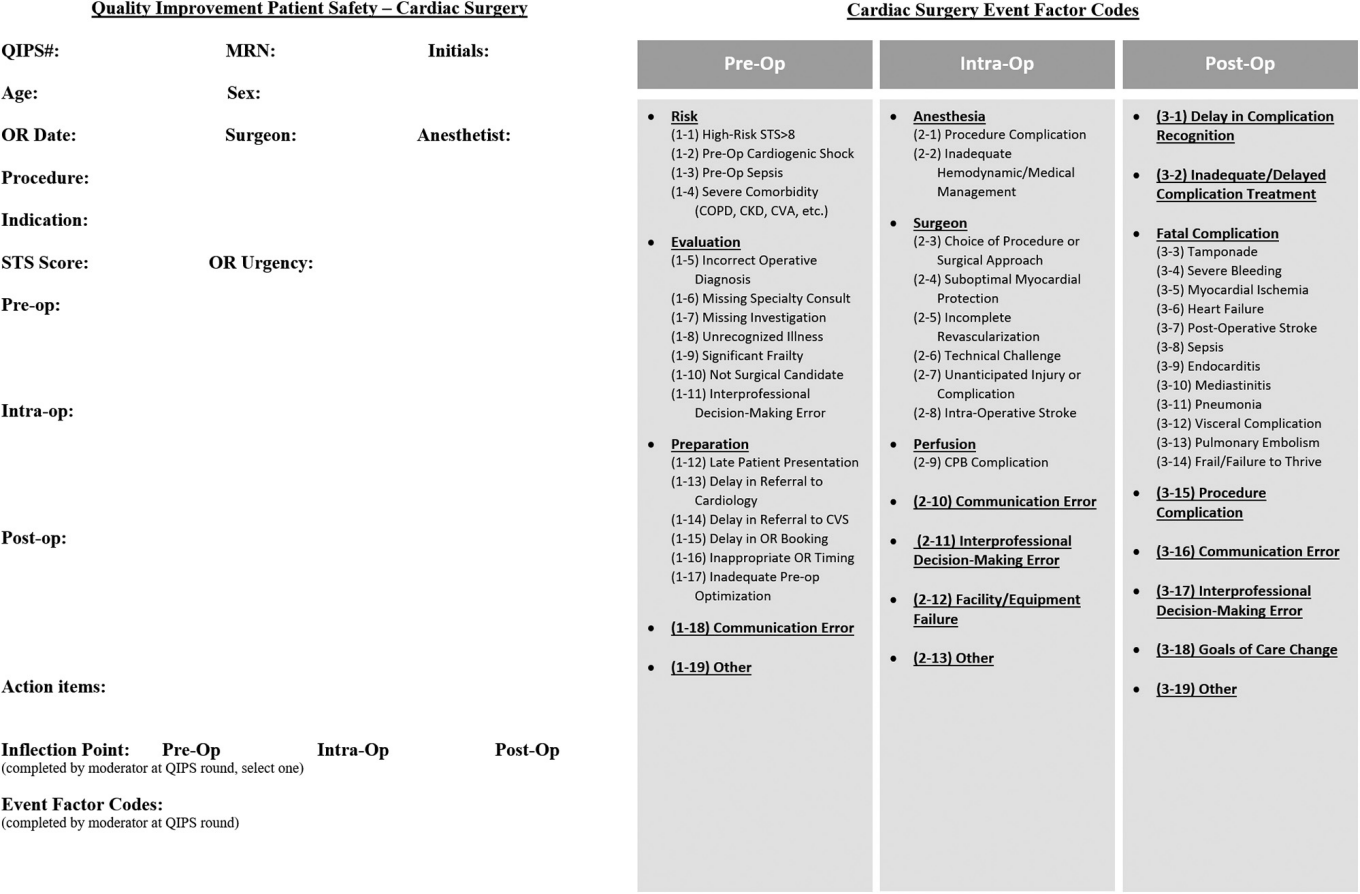
Cardiac Surgery Quality Improvement and Patient Safety (QIPS) Data Sheet. (CKD, chronic kidney disease; COPD, chronic obstructive pulmonary disease; CPB, cardiopulmonary bypass; CVA, cerebral vascular accident; CVS, cardiovascular surgery; Intra-Op, intraoperative; MRN, medical record number; OR, operating room; STS, Society of Thoracic Surgeons, Pre-op, preoperative; Post-op, postoperative.)

**Figure 3:**
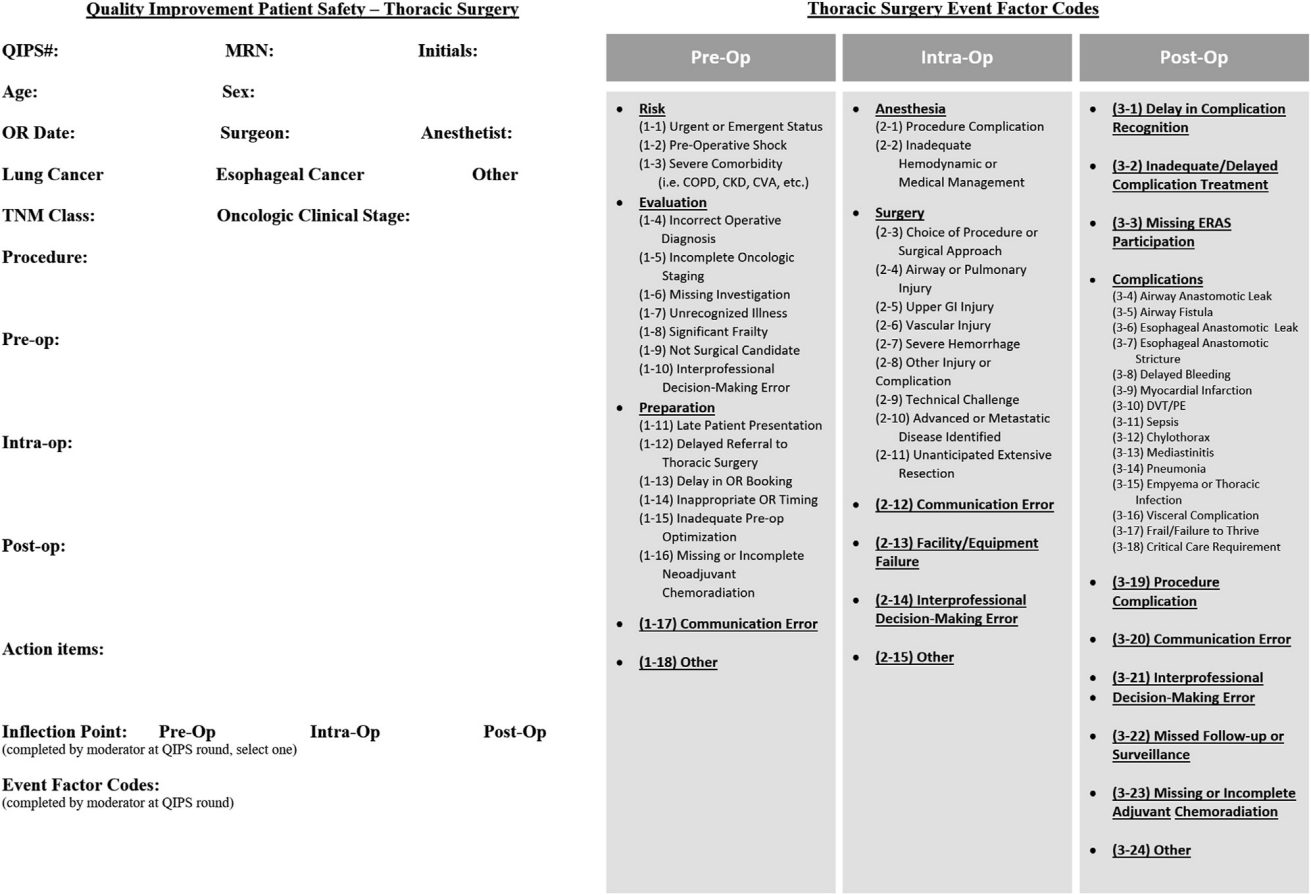
Thoracic Surgery Quality Improvement and Patient Safety (QIPS) Data Sheet. (CKD, chronic kidney disease; COPD, chronic obstructive pulmonary disease; CVA, cerebral vascular accident; DVT, deep vein thrombosis; ERAS, enhanced recovery after surgery; Intra-Op, intraoperative; MRN, medical record number; OR, operating room; PE, pulmonary embolism; Pre-op, preoperative; Post-op, postoperative;TNM, tumor node metastasis.)

Each QIPS case is presented by the individual who was most directly involved in the care of the patient being reviewed. In academic settings, this is usually the resident or fellow who assisted in the primary operation, although if that individual is not available, another trainee should be assigned to present the case. In nonacademic practice, this could be the attending or allied health professional. The cases to be presented are distributed by the program administrator 1 month before the rounds to allow adequate preparation and reflection. Furthermore, the presenter submits a completed QIPS data sheet (see Figure 2 for cardiac data sheet and Figure 3 for thoracic data sheet examples) to the program administrator before the QIPS rounds. The data sheet and case presentation are based on a phase-of-care analysis—preoperative, intraoperative, and postoperative phases—championed by leaders in cardiothoracic quality of care.^5^^,^^8^ Two key deliverables of each case presentation should include “event factors,” issues in the case that could have contributed to the adverse outcome, and the “inflection point,” which is the phase of care when there is an obvious change in the patient’s clinical course trajectory. Each case can have multiple event factors but only one inflection point, which may or may not be directly related. The presenter and attending surgeons should discuss the case to find alignment on event factors and the inflection point before the rounds (Figure 2, Figure 3).

**Figure 4:**
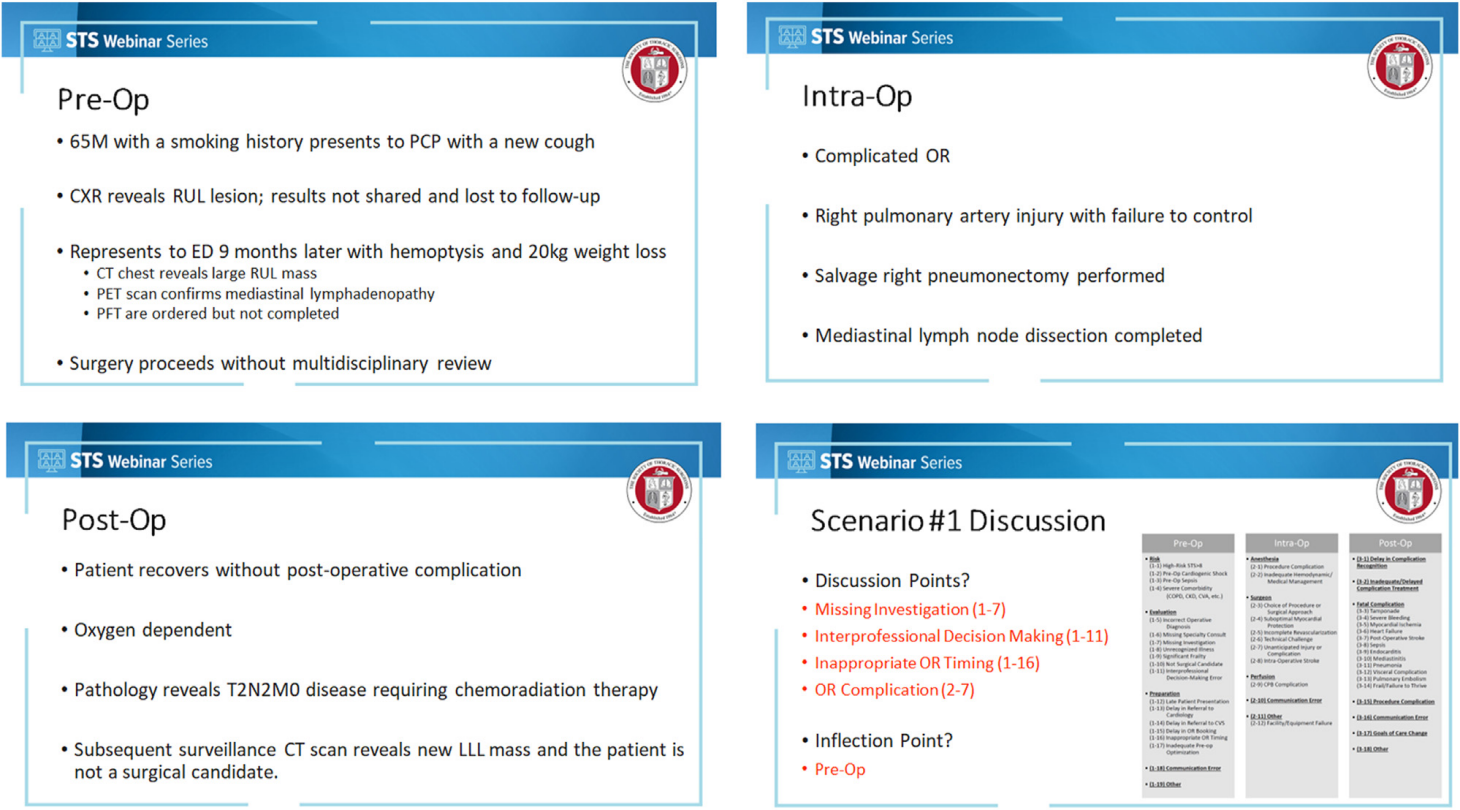
Quality Improvement and Patient Safety (QIPS) case presentation example. The Society of Thoracic Surgeons (STS) logo/Webinar Series template used with permission of The Society of Thoracic Surgeons; all rights reserved (CT, computed tomography; CXR, chest radiography; ED, emergency department; LLL, left lower lobe; OR, operating room; PCP, primary care physician; PET, positron emission tomography; PFT, pulmonary function tests; Post-op, postoperative; Pre-op, preoperative, RUL, right upper lobe.)

The case presentations are a concise description of the clinical course. It is highly beneficial to include significant radiographic images (such as a coronary angiogram, echocardiogram, or chest computed tomography scan) as part of the discussion to help the team further contextualize the clinical case. The presentation closes with the event factors and the inflection point. The attending surgeon should follow with any commentary to allow their point of view, what difficulties they faced through the case, and any constructive critique on the presentation. Overall, the case presentation should be no more than 5 to 10 minutes. See Figure 4 for an example of a QIPS presentation.

The event factors launch the multidisciplinary team discussion, which should focus on confirming or modifying the presenter’s assessment. The discussion between team members should not be derogatory or shameful but provide a safe environment for thoughtful debate, sometimes disagreement, and ultimately distinguish areas for quality of care improvement. The moderator will record the final event factors, inflection point, and any immediate action items on the original case data sheet for input into the QIPS database by the program data manager or submission to relevant administrators. In most jurisdictions, legislation protects documentation related to quality of care reviews (ie, Quality of Care Information Protection Act 2016 in Ontario). Therefore, the details and proceedings of QIPS reviews are kept confidential within the cardiothoracic division.

### QIPS Data Collection and Analysis

The QIPS program is a data-generating process that directly identifies items requiring quality improvement initiatives specific to the cardiothoracic program. The data include patient demographics, operative procedure, operative risk assessment (ie, STS Risk Score or EuroSCORE II), event factors, inflection point, and any immediate action items. A commitment to the QIPS framework yields ongoing growth of the data set and therefore contributes to identifying critical issues for quality improvement.

Because only patients with near-miss, morbidity, or mortality events are captured in QIPS rounds, the analysis is limited to a general descriptive analysis of the patient demographics, operative procedures, event factors, and inflection points. QIPS data and analysis represent a “bottom-up” or “root-cause” approach that is complementary to a “top-down” reporting of clinical outcomes by institutional or STS database. These are complementary analyses as the clinical outcome reporting can show “which” performance markers are below expectations (ie, sternal wound infection after isolated coronary artery bypass grafting or mortality after pneumonectomy for advanced non-small cell lung cancer), whereas the QIPS database looks to identify “why” performance is lagging and possible areas to improve quality (ie, lack of sternal wound infection prevention bundle or selection of prohibitive-risk patient for pneumonectomy).

### QIPS Quality Improvement Initiative Origination

The event factors in the QIPS framework correspond to Donabedian’s quality model of structure (the health care system and resources available) and process (health care professional decision-making and delivery of care) that align with the patient’s outcome.^12^ There is a unidirectional relationship from structure to process to patient outcome, and by categorization of information in this fashion, quality programs can draw inferences on what aspects of health care require attention and improvement. Structural event factors are most prominent in the preoperative phase (late patient presentation, missing diagnostic investigation, delay in surgical consultation, or delay in operating room booking). In contrast, process event factors are present throughout all 3 phases of care (incorrect operative diagnosis, inadequate preoperative optimization, choice of procedure or surgical approach, delay in recognition or treatment of postoperative complication).

The results of the QIPS data analysis will identify one or more patient populations (ie, patients with prosthetic aortic valve infective endocarditis) or event factors corresponding to a structural (ie, interprofessional decision-making) or process (ie, incomplete myocardial revascularization) component as areas for quality improvement that are specific to the cardiothoracic program. QIPS is not a quality improvement initiative itself but rather a systematic framework to identify areas for improvement in cardiothoracic surgical practice. Several well-developed frameworks for quality improvement initiatives, such as that from the Canadian Patient Safety Institute, exist to launch targeted initiatives that address issues highlighted through the QIPS program and process.^13^

## Results

The QIPS framework was presented by the STS Workforce on Patient Safety to members of the STS through webinar sessions in December 2021 and August 2022. Each webinar featured an introduction and description of the QIPS methods followed by 2 mock cases. The cases were presented by pairs of cardiothoracic trainees and attending surgeons who concisely described the clinical proceedings of each morbidity or mortality, followed by identification of the inflection point and event factors and a multidisciplinary discussion by panel members and attendees. The December 2021 webinar had 75 unique viewers from 13 countries and 343 asynchronous playbacks. The August 2022 webinar had 38 unique viewers from 9 countries and 310 asynchronous playbacks.

## Comment

QIPS was designed using the underlying theory, which enhances the effectiveness of quality improvement initiatives, including a well-defined structure and methodology, involving all multidisciplinary stakeholders, consistent selection of cases to be reviewed, goal-directed discussion (event factors and inflection point), critical evaluation, focus on individual and system factors (process and structure), and granular data analysis that yields quality improvement initiatives to facilitate systematic change.^2^^,^^14^ Event factors are the underlying root causes of adverse events and are validated by the multidisciplinary team as part of the data collection process. Over time, through the consistent collection of patient demographics, event factors, and inflection points, a cardiothoracic program’s areas of improvement and targets for quality improvement initiatives can be identified. Notably, institutions can modify the data sheets to include variables that are most important to their programs and even seek alignment with STS event definitions. The data derived from each case review are granular, which holds considerable value over aggregate surgical outcome reporting.^8^ Therefore, QIPS addresses and overcomes several weaknesses of traditional M&M rounds, including heterogeneous program structure, inconsistent impact on quality of patient care, variable participation by attending surgeons, and undertones of shame or fear of disciplinary action related to adverse patient events.

Cardiothoracic trainees are ideal candidates to lead QIPS rounds under the supervision of their attending mentors as they often serve as core members of the clinical team.^15^ They are at the forefront of all 3 patient care phases, interact with all multidisciplinary team members, help manage adverse events, and are therefore best able to hypothesize the potential factors responsible and to synthesize possible solutions.^15^ Trainee-led and attending-supported cases and discussions in a culturally safe and constructive forum are critical to their development as health care practitioners dedicated to quality and excellence in clinical practice.^9^ Ongoing involvement in QIPS and the resulting quality improvement initiatives throughout the spectrum of training are directly in line with the Accreditation Council for Graduate Medical Education and the Royal College of Physicians and Surgeons of Canada mandates for residents to acquire the opportunity, skills, habits, and commitment to continuously pursue quality improvement and patient safety at the individual, program, and institutional level.^9^^,^^10^ Furthermore, the collaboration between the trainee and attending surgeon or multiple responsible surgeons allows an invaluable opportunity for attendings to model professionalism and patient-focused mentalities in a psychologically safe environment.

Whereas the QIPS process was developed in an academic setting, with emphasis on the resident experience, it is also applicable to nonacademic institutions. For example, in the community hospital setting where assistance is provided by midlevel or advanced practice providers, they could present the cases with moderation from attending surgeons. Attending cardiothoracic surgeons might also benefit from analyzing and presenting their own cases to acquire feedback from their partners and the multidisciplinary team. The objective remains to create a multidisciplinary environment focused on a standardized procedure, collaboration and quality, and process improvement rather than the traditional punitive objectives.

In conclusion, QIPS is a new paradigm in cardiothoracic surgical quality. The standardized methods are simple, effective, reproducible, and sustainable. The format is based on Donabedian’s quality model and the phase-of-care analysis methods; it incorporates an invaluable opportunity for cardiothoracic practitioners to develop professional skills, to enhance critical thinking, and to practice quality improvement theory. Fundamentally, when health care practitioners are focused on improving outcomes and can positively respond to constructive comments to optimize practice patterns based on unbiased review and recording of adverse event root causes, cardiothoracic surgical departments directly dedicate themselves to the highest level of quality of care and patient safety imperative to achieving clinical excellence.
